# The extent of ribosome queuing in budding yeast

**DOI:** 10.1371/journal.pcbi.1005951

**Published:** 2018-01-29

**Authors:** Alon Diament, Anna Feldman, Elisheva Schochet, Martin Kupiec, Yoav Arava, Tamir Tuller

**Affiliations:** 1 Biomedical Engineering Dept., Tel Aviv University, Tel Aviv, Israel; 2 The Blavatnik School of Computer Science, Tel Aviv University, Tel Aviv, Israel; 3 Dept. of Molecular Microbiology and Biotechnology, Tel Aviv University, Tel Aviv, Israel; 4 Biology Dept., Technion-Israel Institute of Technology, Haifa, Israel; 5 The Sagol School of Neuroscience, Tel Aviv University, Tel Aviv, Israel; University of Warwick, UNITED KINGDOM

## Abstract

Ribosome queuing is a fundamental phenomenon suggested to be related to topics such as genome evolution, synthetic biology, gene expression regulation, intracellular biophysics, and more. However, this phenomenon hasn't been quantified yet at a genomic level. Nevertheless, methodologies for studying translation (e.g. ribosome footprints) are usually calibrated to capture only single ribosome protected footprints (mRPFs) and thus limited in their ability to detect ribosome queuing. On the other hand, most of the models in the field assume and analyze a certain level of queuing. Here we present an experimental-computational approach for studying ribosome queuing based on sequencing of RNA footprints extracted from pairs of ribosomes (dRPFs) using a modified ribosome profiling protocol. We combine our approach with traditional ribosome profiling to generate a detailed profile of ribosome traffic. The data are analyzed using computational models of translation dynamics. The approach was implemented on the *Saccharomyces cerevisiae* transcriptome. Our data shows that ribosome queuing is more frequent than previously thought: the measured ratio of ribosomes within dRPFs to mRPFs is 0.2–0.35, suggesting that at least one to five translating ribosomes is in a traffic jam; these queued ribosomes cannot be captured by traditional methods. We found that specific regions are enriched with queued ribosomes, such as the 5’-end of ORFs, and regions upstream to mRPF peaks, among others. While queuing is related to higher density of ribosomes on the transcript (characteristic of highly translated genes), we report cases where traffic jams are relatively more severe in lowly expressed genes and possibly even selected for. In addition, our analysis demonstrates that higher adaptation of the coding region to the intracellular tRNA levels is associated with lower queuing levels. Our analysis also suggests that the *Saccharomyces cerevisiae* transcriptome undergoes selection for eliminating traffic jams. Thus, our proposed approach is an essential tool for high resolution analysis of ribosome traffic during mRNA translation and understanding its evolution.

## Author summary

During translation, multiple ribosomes may translate the same mRNA. The density of ribosomal traffic across the transcript poses several open questions, such as how often a ribosome’s path is blocked by a second ribosome, do queues of multiple ribosomes typically form on mRNAs and what is their effect on the overall translation rate of an mRNA. However, this phenomenon hasn't been quantified yet at a genomic level. Nevertheless, methodologies for monitoring translation are limited in their ability to detect ribosome queuing. On the other hand, most of the models in the field assume and analyze a certain level of queuing. Here we present an experimental-computational approach for studying ribosome queuing based on sequencing of RNA footprints extracted from pairs of adjacent translating ribosomes, and a computational model of translation dynamics. Our data shows that ribosome queuing in *Saccharomyces cerevisiae* is more frequent than previously thought, suggesting that at least one to five translating ribosomes is in a traffic jam; these queued ribosomes cannot be captured by traditional methods. Our analysis also suggests that the *S*. *cerevisiae* transcriptome undergoes selection for eliminating traffic jams, while specific regions and genes may possibly be under selection for increased queuing.

## Introduction

Understanding the dynamics of protein translation is a fundamental question in biology, and has been extensively studied using experimental and computational methods in recent years [1,2]. During translation, multiple ribosomes may translate the same mRNA. The density of ribosomal traffic across the transcript poses several open questions, such as how often a ribosome’s path is blocked by a second ribosome, do queues of multiple ribosomes typically form on mRNAs and what is their effect on the overall translation rate of an mRNA.

Computational and mathematical modeling of translation dynamics was first proposed in the 1960s [3–8]. A fundamental aspect of these models is ribosome queuing. Specifically, the totally asymmetric simple exclusion process (TASEP) and variants thereof have been widely used to study ribosome traffic [2,9]. This model assumes that the movement of the ribosomes is only in one direction, from the 5′ end to the 3′ end, a ribosome can only hop to the next position on the transcript at some known rate, and only if it is free from other ribosomes. Thus, it enables a direct study of ribosome queuing. In an early study, Zhang et al. studied the effect of clusters of slowly translated codons on the forming of queues across a synthetic transcript, as well as a set of endogenous mRNAs [10]. Mitarai et al. approximated codon translation rate using 3 categories and showed that ribosome collisions in the *E*. *coli lacZ* operon are more frequent than previously thought, where about 5% of ribosome translation time is wasted because of collisions along the mRNA [11]. In a later study, Mitarai and Pedersen compared the ribosome traffic on 87 wild-type sequences and sequences where the synonymous codons were swapped randomly, and showed that wild-type genes tend to reduce ribosome collisions [12]. Chu et al. performed two studies, accompanied by experiments expressing CFLuc, that analyzed ribosome traffic under different codon sequence variants, and demonstrated the effect of codon usage and queuing of ribosomes in the 5’-end region on translation initiation [13,14]. In recent years, dozens of additional studies based on variants of the TASEP model have been employed to answer fundamental questions in molecular evolution, functional genomics, synthetic biology, and more [2]. However, performing an accurate transcriptome-wide assessment of ribosome queuing remains a challenge due to the need for careful calibration of the model [2].

A number of studies in recent years have suggested that translation is rate-limited by the initiation rate of a gene [15–17]. In this regime, traffic jams are less likely to form due to the low density of ribosomes on the mRNA, and are expected to have a weaker relation with codon usage [18]. However, an ongoing debate remains over the relative contribution of different factors to the control over translation rate, which is also related to the rate limiting nature of initiation vs. elongation [14,19–22].

In recent years, ribosome profiling (Ribo-seq) has been the state-of-the-art technology for monitoring the transcriptome-wide distribution of translating ribosomes at high-resolution [23]. Ribosome profiling is based on deep-sequencing of ribosome-protected mRNA fragments (RPFs) from living cells, such that the sequence of each fragment indicates the position (footprint) of a translating ribosome on the transcript. Ribo-seq has been used to detect positions with significantly enriched reads. Strong peaks in the reads density have been interpreted as ribosome pausing sites and were linked to various possible causes [24–32]. If indeed ribosome pausing is common, we expect queues to form around such pauses.

It should be noted that typically, Ribo-seq protocols filter fragments that are longer than a single ribosome (approximately 30bp), with few exceptions [33]. Thus, adjacent ribosomes and regions where pairs (or more) of ribosomes are frequent, are expected to be under-represented in most Ribo-seq datasets. This may limit the analysis of ribosome stalling and queuing if indeed a significant portion of ribosomes are paired. However, there are currently no comprehensive estimates of the extent of ribosome queuing. In this study, we provide for the first time, quantitative estimates for the portion of pairs of ribosomes and analyze the dynamics of ribosome queuing using experimental and simulated data.

## Results

### Estimating the fraction of paired ribosome footprints

The probability that two ribosomes are adjacent to one another is affected by a long list of variables such as the number of ribosomes in the cell, the length and number of translated mRNAs, the initiation rates, the distribution of elongation rates along the mRNA, etc. Thus, it is not trivial to estimate this value without a direct experiment.

**Fig 1:**
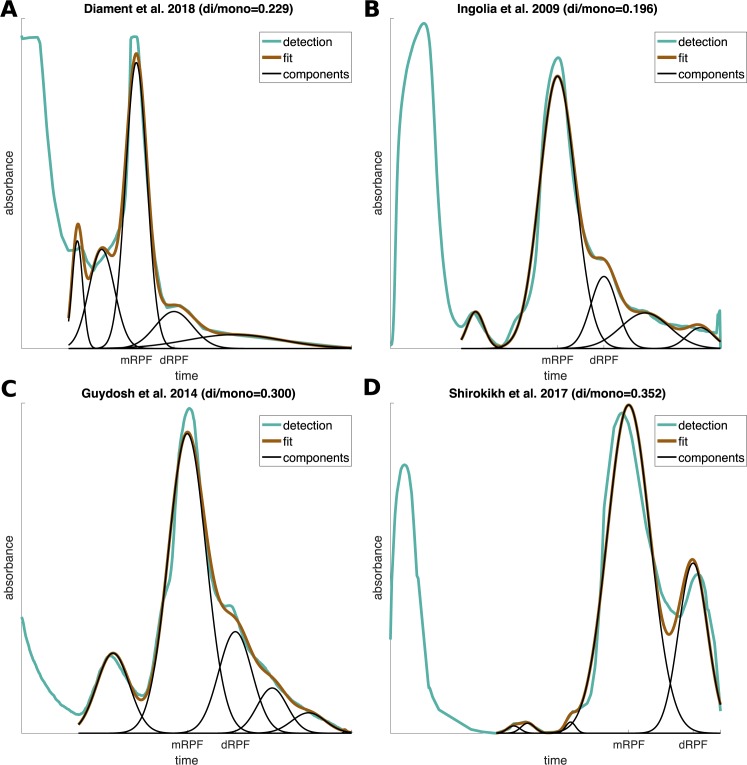
Sucrose gradient fractions. **(A)** Quantification of ribosome protected footprints (RPFs) according to size. The plot shows the estimated components underlying the observed distribution, by means of Gaussian mixture. Each component reflects a set of footprints with a typical size, originating from a single ribosome (mono-RPF, mRPF), a pair of ribosomes (di-RPF, dRPF), etc. The ratio of dRPFs to mRPFs is reported in the caption. **(B)** Same for Ingolia 2009 data, profile reproduced from [23]. **(C)** Same for Guydosh 2014 data, profile reproduced from [33] (Fig 1 in the original paper). **(D)** Same for Shirokikh 2017 data, profile reproduced from [35] (Figure 5 in the original paper). See also **S1 Fig**.

To estimate the quantity of ribosomes that are adjacent to one another in the transcriptome–which is related to the extent of queuing / traffic jams on transcripts–the first steps of the ribosome profiling protocol in yeast were performed as previously described [23,34] (details in the Materials and Methods section). Ribosome protected footprints (RPFs) of various sizes were generated, followed by sucrose gradient fractionation. The resulting profiles were analyzed, along with previously reported footprint gradients [23,33,35], by employing Gaussian mixture to model the different components within the profile (**Fig 1**, details in the Materials and Methods section). Previous studies focused mostly on isolating the RPF fraction with footprints protected by a single ribosome (referred to here as mono-RPFs), with some exceptions [33]. Here, we estimated the size of two fractions: mono-RPFs (mRPFs) and di-RPFs (dRPFs), the latter containing footprints protected by two ribosomes (a disome). The area of each fraction in the profile is proportional to the number of ribosomes in each fraction. Thus, the ratio between the dRPFs fraction and the mRPFs fraction can provide an estimation for the ratio between the number of ribosomes that are directly adjacent to one another and the number of isolated ribosomes on the transcript. The ratio obtained ranged from 0.196 to 0.352 in the four datasets, suggesting that at least 1 in 6 ribosomes is adjacent to another and form a pair. When considering the existence of larger clusters of ribosomes, however, in smaller quantities, the probability of observing a pair of adjacent ribosomes is expected to be even higher, at least 1 in 5 as justified by the computational model in the next sections. We obtained similar results using different methods (**S1 Fig**).

The variance between different studies may reflect the condition in which the experiment was performed, such as the type and amount of RNase used (which may cut dRPFs at some rate and relocate them to the mRPF fraction), but possibly also additional conditions that affect translational efficiency in cells. The high fraction of dRPFs detected in all the experiments, however, suggests that ribosome queuing is more prominent and widespread than typically assumed, and that the distribution and determinants of traffic jams in the transcriptome are important fundamental questions. This may also suggest that due to the large extent of queuing, previous results that attempted to answer these questions by sequencing only mRPFs were based on limited data.

### Measurement of queues

**Fig 2:**
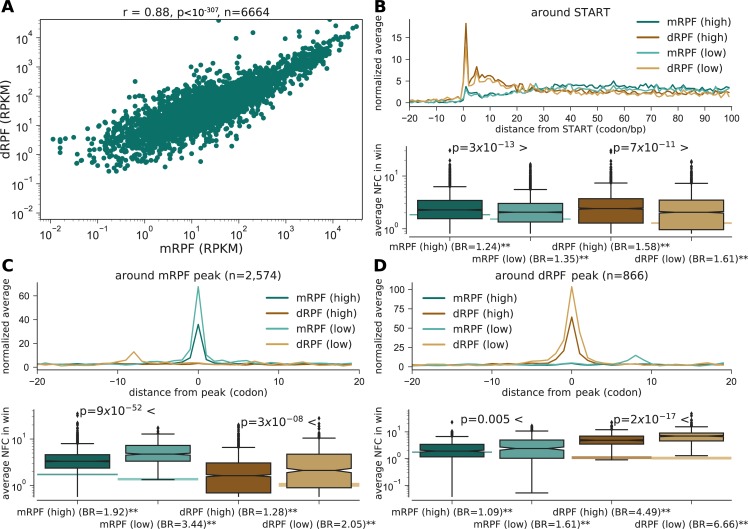
Ribosome profiling. **(A)** mRPFs vs. dRPFs, RPKM values for each gene. Spearman’s rho and an asymptotic p-value are reported in the caption. **(B)** *Top*: Meta-gene analysis around the beginning of the ORF for mRPFs and dRPFs, plotted for highly and lowly expressed genes. Read counts were normalized per gene according to gene average (NFC), and the average across genes per position is shown. *Bottom*: The distribution for all genes of the mean NFC across the window in the top panel. P-values according to rank-sum test compare highly and lowly expressed genes. The background bands show the resulting medians when sampling random positions across the same genes (95% of the sampled medians fall within this range). Background ratio (BR) of the observed median vs. null is reported below (** denotes empirical p-value < 0.01). **(C)** Same, around peaks detected in mRPF profiles. **(D)** Same, around peaks detected in dRPF profiles. See also **S2 Fig**, **S3 Fig**.

In order to study the locations where queues form, we sequenced both fractions of mRPFs and dRPFs (details in the Materials and Methods section). Next, the distribution of mRPFs and dRPFs in the transcriptome was analyzed using our data and Guydosh and Green’s dataset [33]. RPKM (reads per kilobase per million) values of genes according to both RPF types showed a strong correlation between the two (r = 0.88, p<10^−307^, n = 6,664; Spearman’s rho and its asymptotic p-value; **Fig 2A**), however still lower than typically observed between replicates of the mRPF fraction [36]. We expect differences between the distribution of mRPFs and dRPFs to reflect the location of ribosomal traffic jams.

Meta-gene profiles of normalized footprint counts (NFC, i.e. read counts divided by the transcript’s mean read count) were calculated for mRPFs and dRPFs (**Fig 2**, **S2 Fig** and **S3 Fig** for the separate datasets). When examining the 5’-end of genes (**Fig 2B**), an enrichment of dRPFs compared to mRPFs was observed. In addition, it can be seen that the profile of dRPFs decreases into the gene. Genes were divided into two sets of highly and lowly expressed (top / bottom 50% according to their protein abundance). In order to control for the number of reads in the sets, the highly expressed genes were downsampled to have the same total number of reads as the lowly expressed genes. We found that both mRPF and dRPF profiles had higher normalized counts in the set of highly expressed genes compared with lowly expressed genes (p = 3x10^-13^ / 7x10^-11^, respectively, rank-sum p-value). Both mRPFs and dRPFs had a median normalized count significantly higher than the background (1.24–1.35-fold for mRPFs and 1.58–1.61-fold for dRPFs, p<0.01, empirical p-value based on random sampling of positions within the same set of genes). This may imply that ribosome queuing is frequent in the 5’-end region, and specifically in highly expressed genes.

Peak calling was performed in order to detect regions with significant ribosome pausing, and study its association with the traffic around the peak. Peaks were defined as positions that are at least 5 standard deviations above the ORF’s mean (ignoring positions empty of reads). In the vicinity of detected peaks in the mRPF profiles a dRPF peak can be observed, distanced 9 codons upstream (**Fig 2C**). These di-footprints mark the position of two ribosomes–a ribosome positioned 9 codons upstream to the mRPF peak, and a second one approximately where the mRPF was detected. Unlike in the 5’-end region, mRPF peaks were more extreme in lowly expressed genes (p = 9x10^-52^, 3.44-fold above background compared with 1.92 in top genes). Similarly, the associated dRPF peaks were mostly related to lowly expressed genes (p = 3x10^-8^, 2.05-fold above background compared with 1.28). Correspondingly, in the vicinity of peaks detected in dRPF profies an mRPF peak was detected 9 codons downstream (**Fig 2D**). Furthermore, the dRPF peaks were more extreme in lowly expressed genes (p = 2x10^-17^, 6.66-fold above background compared with 4.49), and the associated mRPF reads were enriched in lowly expressed genes (p = 0.005, 1.61-fold above background compared with 1.09). One hypothesis that may explain this result is that when traffic jams form in lowly expressed genes, they tend to be more severe. Traffic jams are expected to form in regions with high ribosomal density–for example, in highly expressed genes with high initiation and translation rates. On the other hands, highly expressed genes are under selective pressure to increase translation efficiency and to reduce traffic jams, for example by developing a ramp in their 5’-end [37,38]. Due to this, lowly expressed genes may still contribute significantly to the observed queues despite being less dense.

**Fig 3:**
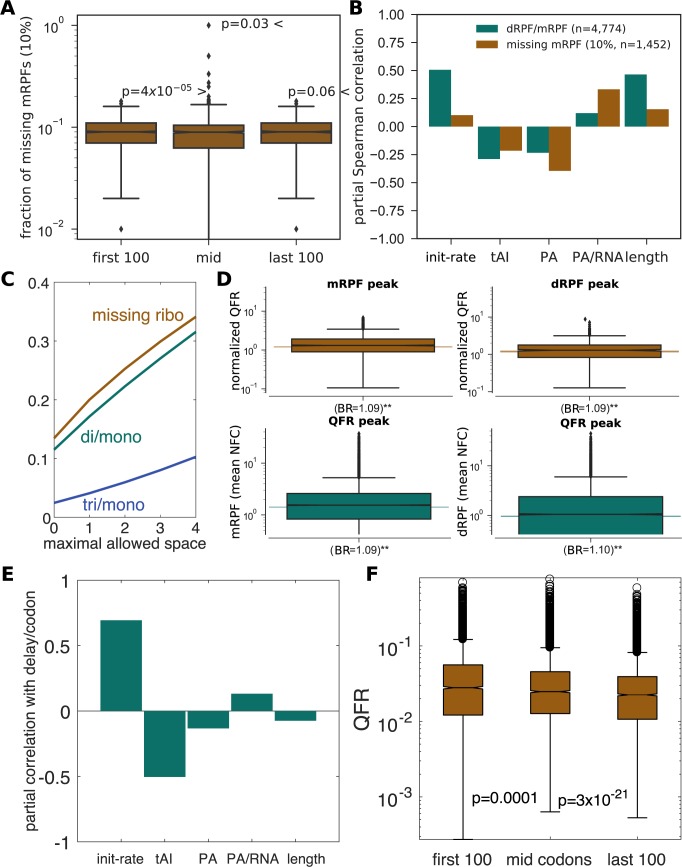
Queue analyses. **(A)** Fraction of positions with detected missing mRPFs in genes, divded into 3 regions: first 100 codons, last 100 codons and the rest of the gene. mRPF profiles were downsampled to have the same number of reads as the dRPF profiles. Rank-sum p-values reported. **(B)** Partial Spearman correlations between the dRPF-to-mRPF ratio per gene, and the fraction of missing mRPFs (across the entire ORF) vs. various features (initiation rate, tRNA adaptation index, protein abundance–PA, PA per RNA copy and ORF length), while controlling for their dependencies. mRPF profiles were downsampled to have the same number of reads as the dRPF profiles. *n* indicates the number of genes, and the threshold for missing mRPF detection is indicated in percentage. See also **S4 Fig**. **(C)** The ratio between simulated dRPFs and mRPFs, as well as the estimated fraction of missing ribosomes when sequencing mRPFs, for different values of the maximal allowed space between ribosomes on a footprint (see also **S4 Fig**). **(D)** *Top*: Enriched simulated queued ribosomes in windows surrounding measured mRPF and dRPF peaks (empirical p-value < 0.01). Y-axis shows the average QFR (normalized similarly to NFC). *Bottom*: Enriched mRPFs and dRPFs in windows surrounding peaks in simulated profiled of the fraction of queued ribosomes per position (empirical p-value < 0.01). **(E)** Partial Spearman correlations between ribosome delay per codon of a gene, and various features while controling for their dependencies. **(F)** Distribution of the queued fraction of ribosomes (QFR) in 3 regions of the gene. Sign-rank p-value between box-plots.

Whenever ribosomes form a queue, the traditional ribosome profiling protocol (which sequences mRPFs) is expected to fail to fully capture the present ribosomal density. We tried to estimate the percentage of queued positions and locations of such queues by detecting missing mRPFs in the profiles. The following rule was employed: a position had a significant number of mRPFs missing if the number of mRPFs in that position was in the bottom 10% of that gene, while the number of dRPFs in that position was in the top 10% of that gene. For each gene with at least 10% dRPF reads coverage, the percentage of positions with missing mRPFs was calculated according to this rule (median: 9%). In addition, the calculation was repeated for 3 regions of the gene: the first 100 codons, the last 100 codons and the rest of the gene (**Fig 3A**). The percentage of positions with missing mRPFs was significantly higher in the first / last regions (p = 4*x*10^-5^ / 0.03, respectively; rank-sum p-value), compared with the mid-region, suggesting that these regions tend to contain more traffic jams.

Finally, the correlation between the percentage of positions with missing mRPFs in a gene and various features related to its expression and translational efficiency was computed, while controlling for dependencies between the features using partial Spearman correlations. Significant negative correlations were observed with the gene’s adaptation of the coding sequence to the tRNA pool as measured via tAI [39] (r = -0.21, p = 1.4x10^-6^), and its protein abundance (PA, r = -0.39, p = 3x10^-55^). Significant positive correlations were observed with the initiation rate of the gene (r = 0.10, p = 10^−4^), the PA per RNA copy (r = 0.33, p = 8x10^-39^), and ORF length (r = 0.15, p = 2x10^-9^; **Fig 3B**). Initiation rates for the aforementioned test were inferred using a simulation of ribosome traffic, as discussed in the next section and the Materials and Methods, with ribosomal densities and RNA copies estimated from Ribo-seq and RNA-seq data. Similar results were observed using different thresholds for detection of missing mRPF and appear in **S4 Fig**. The test was repeated for the dRPF-to-mRPF ratio with similar results (**Fig 3B**). We further discuss these results below.

### Translation simulation

Simulating ribosome movement enables a study of traffic on the mRNA in greater detail. A simulation of translation in 5,450 yeast genes was performed based on the totally asymmetric simple exclusion process (TASEP) model, with parameters calibrated using Arava et al.’s experimental measurements of the number of ribosomes on each transcript as well as RNA copy numbers [40] (details in the Material and Methods section, simulation results reported in **S1 Table**). Translation initiation rates of genes were fitted to achieve the measured ribosomal density. In addition, RNA and ribosome numbers were scaled globally to achieve the total quantities of transcripts and translating ribosomes that are generally accepted in the literature (details in the Materials and Methods section). The resulting initiation rates (median: 0.09s^-1^, with rates ranging from 0.019s^-1^ in the 10^th^ percentile to 0.29s^-1^ in the 90^th^ percentile) were in good agreement with previous results (r = 0.70, p<10^−307^, n = 5,162; asymptotic p-value for Spearman’s rho) [41]. The correlation between the resulting translation rates and PA per RNA copy was moderate (r = 0.32, p = 2.2x10^-16^, n = 5,132), and similar to their correlation with the ratio of Ribo-seq RPKM to RNA-seq RPKM (r = 0.37, p = 1.3x10^-170^, n = 5,384). To test the simulated profiles at the codon level, density profiles of the 1,000 densest genes in a high-resolution ribosome profiling dataset were utilized [42]. A median correlation of 0.10 was observed between simulated and measured ribosome density profiles of genes, smoothed using a 10-codon wide sliding window. This value is comparable to the correlation between two mRPF profiles from different experiments (r = 0.16 for Brar et al. vs. Guydosh et al.; this is similar to previous reports [36]).

We generated a simulated distribution of the fractions of RPFs in the simulation (ribosomes were taken to be 10 codons long), counting the number of isolated, pairs, triplets of ribosomes etc. throughout the transcriptome (**S4 Fig**). First, only directly adjacent ribosomes were considered, with no codons between them, resulting in a dRPF-to-mRPF ratio of 0.11, which is lower than reported in the section above. However, when considering simulated RPFs comprising of ribosomes up to 1–4 codons apart on the footprint, the observed ratio increased to 0.17–0.32 –results that are in good agreement with the ones above (**Fig 3C**). Thus, it is possible that some of the RPFs that are typically collected in a ribosome profiling experiment contain ribosomes with 1–4 free codons between them. Other parameters that may affect this ratio are the growth conditions, and the existence and frequency of ribosome pausing, which was not simulated here [25]. Similarly, the simulation was utilized to estimate the size of the fraction of tri-RPFs (containing 3 ribosomes), which was 3- to 5-times smaller than the dRPF fraction, suggesting that dRPFs represent the majority of queued ribosomes (**Fig 3C**, **S4 Fig**). Moreover, the model enables to extrapolate and estimate the fraction of total ribosomes that are not isolated since they are adjacent an additional ribosome, and therefore are unlikely to be detected via mRPF sequencing. We estimate that this fraction ranges from 14% (0 codon separation between neighboring ribosomes) to 34% (up to 4 codon separation) (**Fig 3C**).

### Simulated queue properties

We analyzed the distribution across the transcriptome of queued ribosomes and their waiting times in the simulation, that is, ribosomes that cannot complete an elongation cycle due to the next codon being covered by another ribosome. The probability of observing a queue in a gene was 0.21 (median over genes, with probabilities ranging from 0.024 in the 10^th^ percentile to 0.58 in the 90^th^ percentile). Typically, 3.67% of the translating ribosomes on an mRNA were delayed due to traffic jams (median over genes, with the 10^th^ and 90^th^ percentiles being 0.9% and 14.2%), and 5.9% of all translating ribosomes in the cell were delayed at any given time. This fraction is related to the number of observed dRPFs in the previous section, however it is expected to be smaller than the latter since some of the ribosomes that are within a footprint’s range from one another may not interfere with another ribosome’s progress at all times (only at time intervals when the two are directly adjacent and the downstream ribosome is elongating more slowly than the upstream ribosome). The total delay time of a ribosome during the synthesis of a complete gene was 3.5s (median over genes, with delay times ranging from 1.07s in the 10^th^ percentile to 11.65s in the 90^th^ percentile).

Next, the meta-gene analyses in the above section were repeated for simulated profiles. In windows surrounding observed Ribo-seq peaks (the ones detected in **Fig 2**), a significant enrichment was observed in the queued fraction of (simulated) ribosomes (QFR) (1.09-fold compared with background around mRPF and dRPF peaks, **Fig 3D**). Similarly, peaks were detected in the profiles of the QFR values per position, with significant enrichment of mRPF and dRPF reads observed in their vicinity (**Fig 3D**). Thus, the simulated traffic has a significant relation with the measured RPFs. Differences between the experimental data and simulation can be partially explained by the high level of inter-experiment variance, and by the fact that our model does not include long translational pauses, which may affect a significant portion of ribosomal traffic [25].

We tested the relation between ribosome delay times per codon and various sequence/expression-related features of the gene, including the inferred initiation rate, tRNA Adaptaion Index (tAI) score, protein abundance (PA), PA per RNA copy, and ORF length. Partial Spearman correlations were computed between ribosome delay times per codon and each feature given the other 4 features (**Fig 3E**). A strong positive correlation with initiation rates (r = 0.69; p<10^−307^; n = 5,080; asymptotic p-value) and a strong negative correlation with tAI scores (r = -0.50, p<10^−307^, n = 5,080) were observed, implying that genes that contain codons recognized by less abundant tRNA species tend to exhibit ribosome congestion and traffic jams across their sequence. These results are similar to the ones reported above based on Ribo-seq data (**Fig 3B**). Furthermore, weak yet significant correlations were observed with PA (r = -0.13; p = 1.4x10^-21^; n = 5080), PA/RNA (r = 0.13; p = 3.2x10^-32^; n = 5,080) and gene length (r = -0.07; p = 10^−7^; n = 5,080). Next, QFR was computed in 3 different regions of the ORF–the first 100 codons (median: 2.8%), the last 100 codons (2.25%) and the rest of the sequence (2.48%)–for 4,204 genes with appropriate length (**Fig 3F**). On average, the percentage of queued ribosomes in the first region was 1.9/2.1-fold higher in the first region compared with the middle/last region (respectively) in that gene; this is with agreement with the 5’-end enrichment observed in Ribo-seq data.

The simulation was repeated for 100 random genomes where the order of synonymous codons within each gene was permuted, as well as for 100 random genomes where the global rates of codons were shuffled (without altering the gene sequence, details in the Material and Methods section), and employed them to calculate two empirical p-values: p_1_ controlling for codon order, and p_2_ controlling for the general decoding rates of the different codons. We found that traffic jams were less prevalent in the real genome according to the various properties discussed above, including: ribosome delay time (p_1_<0.01, p_2_<0.01), queue probability (p_1_<0.01, p_2_ = 0.01) and QFR (p_1_<0.01, p_2_<0.01), as well as the ratio of simulated dRPF to mRPF (p_1_<0.01, p_2_<0.01). Interestingly, the percentage of queued ribosomes in the first 100 codons was higher than in permuted sequences (p_1_<0.01), while in other regions of the gene it was lower than in permutations (p_1_<0.01). This is in agreement with previous suggestions that selection acts on codon order to decrease congestion downstream by delaying ribosomes when entering the ORF [38].

P_2_ values were also utilized to test the simulation calibration. The simulation results were in better agreement with experimental data for the selected set of codon decoding rates than for random assignments of the same 61 rates. For example, the correlation between simulated and measured footprint density profiles was significantly higher for the selected parameters (p_2_<0.01), as was the correlation between simulated translation rates and PA/RNA (p_2_ = 0.01), and the correlation between simulated translation rates and Ribo-seq/RNA-seq RPKM ratio (p_2_ = 0.01).

### Functional gene groups with extreme traffic conditions

**Fig 4:**
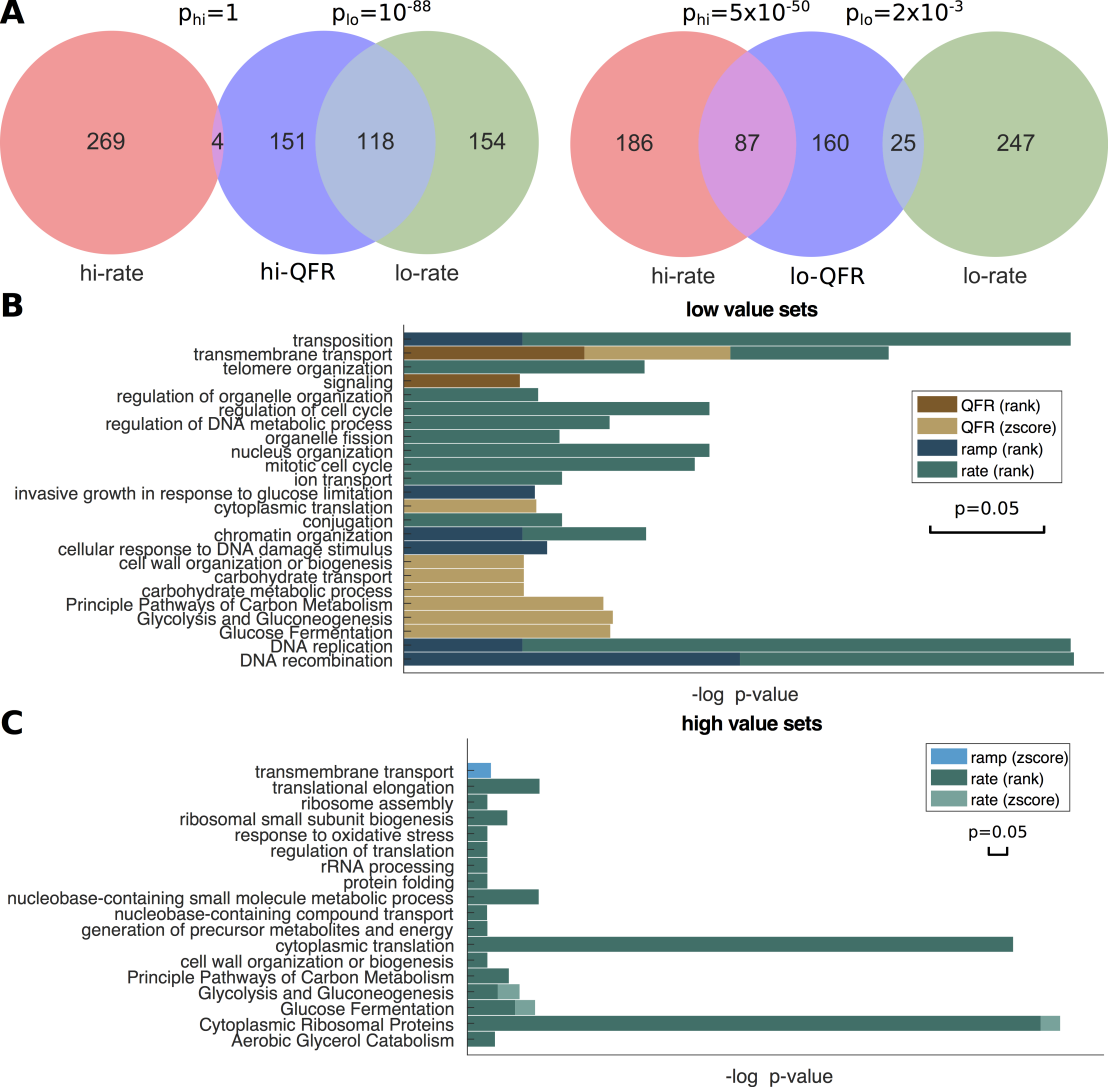
Functional enrichment. **(A)** Sets of genes with extreme properties relative to randomizations of their synonymous codon order. *Left*: Intersection between the set of genes with the highest QFR compared to random, and genes with the highest/lowest translation rate compared to random. *Right*: The same for the lowest QFR compared to random. **(B)-(C)** Functional enrichment in different gene sets with extreme properties, according to their rank among genes, or according to their z-scores vs. codon randomizations. Each of the stacked bars represents the enrichment p-value of the corresponding term (longer bars for smaller p-values) in the gene set (denoted by color, see legend). The ramp score was defined by the ratio of QFR in the first 100 codons and the middle of the gene (excluding the first/last 100 codons). The scale of the 0.05-threshold is shown below the legend.

We studied gene sets that exhibited extreme traffic conditions, either with respect to the rest of the genes or with respect to randomized variants of these genes. The resulting z-scores from comparing an observed property of a gene (e.g., its translation rate) with the distribution of that property in variants with shuffled synonymous codons and the same ribosomal load on the transcript can be regarded as an optimality score for the sequence (details in the Materials and Methods section). The set of genes with the highest translation rate compared to its respective random set (top 5%) has a significant subset of common genes with the set of genes with the lowest fraction of queued ribosomes compared to random (bottom 5%, p = 5x10^-50^, hypergeometric p-value), and vice versa (p = 10^−88^, **Fig 4A**). This is expected, as genes with an optimized codon order (and reduced traffic jams) can reach a higher translation rate while occupying the same average number of ribosomes (see for example [37]). In addition, a significant negative correlation was observed between the z-scores of QFR and the inferred initiation rates of genes reported in the sections above (r = -0.13, p = 6.2x10^-21^, n = 5,450). This result is in agreement with the hypothesis that highly expressed genes are expected in many cases to be under selective pressure to optimize their sequence as to minimize queuing (see for example [37]).

Functional enrichment was performed for the above sets of genes (**Fig 4B and 4C**, sets appear in **S2 Table**), detecting a number of biological processes and pathways over-represented in genes with a low QFR z-scores, such as cytoplasmic translation (p = 0.0305, hyper-geometric p-value adjusted for FDR, light brown in **Fig 4B**), and glucose fermentation (p = 0.004, light brown in **Fig 4B**). The largest subset of QFR-optimized genes was associated with transmembrane transport (p = 0.0215, light brown in **Fig 4B**). Interestingly, the same process was also enriched in genes with high z-scores of dense queuing in the first 100 codons compared with the middle codons of the genes (p = 0.0216, light blue in **Fig 4C**). Many of these genes are not highly expressed, as discussed below, and may be selected for these properties for reasons other than translational efficiency, such as co-translational folding [43].

Next, functional enrichment was performed for sets of highly and lowly ranked genes, according to their properties (sets appear in **S2 Table**). A number of pathways were enriched both in highly translated genes (top 5%, dark green in **Fig 4C**), as well as in rate-optimized genes (top 5% of z-scores, light green in **Fig 4C**) according to translation rate, namely cytoplasmic ribosomal proteins (p_rank_ = 2.8x10^-41^, p_zs_ = 0.0422 for the highly ranked and highly optimized sets, respectively), glycolysis (p_rank_ = 7.1x10^-3^, p_zs_ = 0.0285) and glucose fermentation (p_rank_ = 4.2x10^-4^, p_zs_ = 0.0382). A number of pathways were enriched in highly translated genes (dark green in **Fig 4C**), as well as in QFR-optimized genes (light brown in **Fig 4B**), namely cytoplasmic translation (p_rank_ = 2.5x10^-39^, p_zs_ = 0.0305), carbon metabolism (p_rank_ = 1.2x10^-3^, p_zs_ = 5.2x10^-3^), cell wall organization (p_rank_ = 0.0385, p_zs_ = 0.0424), glycolysis (p_zs_ = 4.1x10^-3^) and glucose fermentation (p_zs_ = 4.4x10^-3^). This is expected, as highly translating genes also tend to have higher ribosomal densities and therefore their codon usage may be under selection to decrease traffic jams.

## Discussion

Ribosome traffic jam is a phenomenon that has been mentioned many times in the context of genome evolution, biophysics of translation, translation modeling, biotechnology, and more. To the best of our knowledge, this study provides the first comprehensive experimental quantification of traffic jams during translation. We estimated that at least 20% of the ribosomes in the budding yeast *S*. *cerevisiae* are positioned close enough to another ribosome on the transcript to be collected as a single footprint in a ribosome profiling experiment. This is a direct indication for the level of queuing in transcripts. Our estimates using sucrose gradient analyses have been consistent across experiments, and with simulated ribosome traffic based on empirical parameters. The observed variance in results obtained from different datasets may have been related to the experiment’s particular conditions and protocol. The current experimental resolution limits the accuracy of analysis to footprints containing a single (mRPF) or a pair (dRPF) of ribosomes, but future Ribo-seq experiments may enable the analysis of larger footprints.

The results reported here suggest that queuing is not a negligible phenomenon. It appears to have important effects on organism evolution and fitness. Since translation is the process that consumes most of the energy in the cell [44], an increase of x percentage in ribosome density should be related to a similar increase in growth rate or fitness (as it is directly related to the energy consumed by the cell). It is clear that even a change of 1% or even 0.1% (as was estimated in **Fig 3D**) in the fitness of a micro-organism should have a very strong effect on its evolution (for example, in the case of *S*. *cerevisiae*, an organism with a doubling time of 90min, a mutant with 0.1% increase in fitness is expected to practically be 97% of the population after 8 months). Moreover, increase in traffic jams may have an even stronger impact when considering its potential effect on ribosome abortion, that can be related again to energy waste, as well as to the production of nonfunctional and even toxic truncated proteins. Thus, from a biological/evolutionary point of view these values are potentially significant. We believe that further comprehensive experimental and computational efforts should be spent in the future to better understand and quantify the effect of local increase/decrease in ribosome densities/queues on organism fitness.

Due to these implications, ribosome queuing should be considered when designing Ribo-seq experiments (or using any other technology for studying translation), when analyzing mRNA translation, or when modeling mRNA translation. Specifically, the results reported here support the usage of the dozens of TASEP based models in studies that have been published in recent years [2,9,45]. These results also support the idea that the genomes of various organisms are under selection for generally minimizing queuing (see, for example, [10,38,41,46]).

Our approach has enabled the detection of transcript properties that are associated with high/low levels of queuing; for example, we showed that higher initiation may tend to increase queuing while higher adaptation to the tRNA pool may tend to decrease queuing. Thus, our methodology can be used for developing better models of translation and for designing synthetic genes with increased/decreased queuing levels.

In summary, we believe that the computational-experimental approach demonstrated here will enable the analysis of translation at a higher resolution. It is essential to perform similar studies in various organisms and in different conditions to understand the significance of queuing across the tree of life. In the current study, we focused on translation under standard growth medium for yeast. It is important to emphasize that it is generally believed that under stress condition there are more traffic jams due to non-typical expression of tRNA species [47]; thus, it is possible that the estimates reported here serve as a lower bound for the ribosome traffic levels observed in the environment and evolution of yeast and other micro-organisms.

## Materials and methods

### Ribosome profiling

Ribosome profiling was performed as previously described [23,34], with minor modifications. rRNA was depleted using Ribo-Zero by EpiCentre. The small RNA sequencing kit from New England BioLabs [48] was utilized. Sucrose gradient fractionation was employed for filtering reads containing one or two ribosomes. Additional filtering of DNA was performed with BluePippin to select the relevant length prior to sequencing.

### Ribosome profiling analyses

A Gaussian mixture model was fitted to the sucrose gradient profiles of ribosome footprints. To this end, an iterative expectation-maximization algorithm was used (implemented in MATLAB’s fitgmdist function). As input to the algorithm, 500 equally spaced points were sampled from the X-axis of the profile (which is related to particle size), with their representation in the input set (the number of times each point appears) proportional to their absorbance value (the point of maximum absorbance arbitrarily set to appear 100 times in the input set). Model fitting was run multiple times using a varying number of components (4 to 8), using random seeding of the algorithm, as well as seeding with components centered around peaks in the profile (detected using MATLAB’s findpeaks), until reaching an optimal solution. The quality of models was assessed based on the Bayesian information criterion and manual inspection of candidate solutions with scores distanced at most 0.5% from the optimum.

Transcript sequences of *Saccharomyces cerevisiae* were obtained from Ensembl [49] (R64 release 87), and UTR lengths were based on previously reported major transcript isoforms (mTIF, selecting the longest one available for each transcript, and using a default length of 500bp for missing UTRs) [50]. Adaptors (AGATCGGAAGAGCACACGTCT in our dataset) were trimmed from reads using Cutadapt [51] (version 1.12). Bowtie [52] (version 1.2) was employed to map them to the transcriptome. In the first phase, Ribo-seq reads that mapped to rRNA and tRNA sequences were discarded using Bowtie parameters ‘-n 2—seedlen 21 -k 1—norc’. In the second phase, the remaining Ribo-seq reads, as well as RNA-seq reads, were mapped to the transcriptome with Bowtie parameters ‘-v 2 -a—strata—best—norc -m 200’. When the 3’ adaptor contained polyA alignments were extended to their maximal length by comparing the polyA with the aligned transcript until reaching the maximal allowed error (2 mismatches). Unique alignments were first assigned to the ribosome occupancy profiles. For multiple alignments, the best alignments in terms of number of mismatches were kept. Then, multiple aligned reads were distributed between locations according to the distribution of unique ribosomal reads in the respective surrounding regions. To this end, a 100nt window was used to compute the read count density RCD_*i*_ of unique reads in vicinity of the *M* multiple aligned positions in the transcriptome, and the fraction of a read assigned to each position was ${\mathrm{RCD}}_{i}/\sum_{j=1}^{M}{\mathrm{RCD}}_{j}$. The location of the A-site (the site of the upstream ribosome in the case of dRPFs) was estimated for each read length by performing meta-gene analysis of the distribution of 5’-end of reads upstream to the start codon of ORFs. If a global maximum was detected in the range of 5-20bp upstream to the start codon (no larger peak observed down-/upstream from this range), this distance was selected as the shift to the P-site for this read length, otherwise these reads were discarded. For 2 datasets where peaks were hard to detect, a default shift of 15bp was employed. Mapping statistics and accession numbers for all datasets used appear in **S3 Table** and **S5 Fig**. An aggregated dataset of our experimental data and Guydosh et al.’s was analyzed in **Figs 2 and 3**, and each dataset was analyzed independently in **S2 Fig** and **S3 Fig**. Protein abundance data for dividing genes into highly / lowly expressed sets was obtained from PaxDB (GPM_2012_09) [53]. Ribo-seq reads were analyzed in Python 3.5 using custom scripts and Biopython 1.6.8, SciPy 0.19.0, and NumPy 1.12.1.

### Translation simulation

Translation was modelled stochastically using a totally asymmetric simple exclusion process (TASEP) model, as performed in previous studies [54–56]. An mRNA with *N* codons is modeled as a lattice of *N* sites. At any time *t*, each site can be occupied by a single ribosome. Each ribosome translates a single site, as well as covers *L* sites in total, so that other ribosomes are excluded from these sites (cannot occupy or translate them).

The model dynamics consist of 3 possible events: (1) A free ribosome will attach to site *i* = 1 with rate *λ*, provided that the first *L*/2 codons on the mRNA are empty; (2) An attached ribosome located at site *i* will move to the next site *i* + 1 with rate *λ*_*i*_, provided codon *i* + *L*/2 is not covered by another ribosome (otherwise we refer to this ribosome as “queued” or “delayed”); (3) In the case of i = *N* the ribosome movement is in fact a termination step. It is assumed that the time between initiation attempts is distributed exponentially with rate *λ*. In addition, it is assumed that the time between jump attempts from site *i* to *i* + 1 is exponentially distributed with translation rate *λ*_*i*_, which is inversely proportional to the mean translation time *t*_*i*_ of the codon. It follows, that the time between any consecutive events is exponentially distributed (the minimum of exponentially distributed random variables) with the rate *μ*, given by: $\mu ({\left\{n_{i}\right\}}_{i=1}^{N})=\lambda +\sum_{i=1}^{N}n_{i}\lambda_{i}$, where *n*_*i*_ ∈ {0,1} denotes the translation state of site *i* (equal to 1 while being translated, and 0 otherwise). The probability that a specific event was an initiation attempt is given by *λ*/*μ*({*n*_*i*_}), while the probability that a specific event was a jump attempt (or termination event) from site *i* to site *i* + 1 is given by *n*_*i*_*λ*_*i*_/*μ*({*n*_*i*_}).

In each simulation step, an event and the time that passed between events are sampled according to the above distributions. Next, state variables are updated according to the rules for each event. A pseudo-code of the simulation is given in **S1 Text**. In order to bring the system to a steady state, the simulation was initiated with an mRNA empty of ribosomes, followed by simulation steps until reaching 100 terminations. The system was then simulated for additional 10,000 terminations while keeping track of various properties of the system (details in the Monitored features section below). For example, the steady state rate of protein production was determined by dividing the number of termination events by the total time that has passed. For this number of simulation steps, the difference between two simulation runs in the translation rate for a gene was small, typically 0.6% (median, ranging from 0.1% in the 10^th^ percentile of differences between genes to 2% in the 90^th^ percentile). The simulation was implemented and analyzed in MATLAB R2016b.

### Monitored features

During simulation, steady state distributions were generated for a number of variables related to the translation process. The state variables {*n*_*i*_} were integrated over the simulation time, and divided by total simulation time to get a vector of ribosomal densities for each site on the transcript (the fraction of time a site was occupied by a ribosome). The sum of which is equal to the mean number of ribosomes on the transcript in steady state. Similarly, density profiles were generated for simulated footprints of any feasible size *k* (the number of ribosomes in the footprint) by assigning, at each simulation step, every ribosome to a footprint set according to its distance from neighboring ribosomes. If the distance between two ribosomes was smaller than the maximal allowed spacing, the pair was assigned to the same footprint. After completion of assignments, state variables $\left\{m_{i}^{k}\right\}$ were set to 1 (translated) / 0 (otherwise) according to the position of the upstream ribosome in each footprint of size *k*. The simulated footprint density profiles were obtained by integrating $\left\{m_{i}^{k}\right\}$ over time. Finally, queuing and delay of ribosomes were monitored during the simulation. First, a state variable was defined per site {*d*_*i*_}, which was set to 1 once a ribosome in site *i* failed at a jump event due to the next codon being covered by another ribosome, and was set back to 0 once the ribosome successfully jumped. The density of queued ribosomes was obtained by integration similarly to above; the sum of which gives the average number of queued ribosomes on the transcript (or the queued fraction of ribosomes, QFR, when normalized by the number of ribosomes in the gene/region). Second, the total time that each ribosome spent translating the RNA, as well as the total time spent in queued state, were averaged over the simulated terminations to obtain the average translation and delay times. Finally, a state variable *q* was defined for the transcript, which was set to 1 as long as any ribosome was delayed across the transcript, and utilized it to obtain the probability for a queue.

In order to keep track of the quantities of ribosomes / RPFs in the cell, the RNA copy numbers measured by Arava et al. [40] were employed, while taking into account the estimated occupied (actively translated) RNA copies and the simulated ribosomal / RPF densities described above. For the purpose of computing the correlation between the simulated profiles and Ribo-seq read count profiles (as reported in **S5 Fig**), reads were sampled from this simulated RPF distribution until reaching an equal number of reads to those in the Ribo-seq experiment.

### Simulation calibration

Codon translation times were obtained from [57], where they were fitted using a whole-cell translation simulation model with finite cellular resources and Ribo-seq data (**S4 Table**). Initiation rates were selected to optimize the fit of the number of ribosomes on each transcript in steady state to the experimental data of Arava et al. [40]. In this study, the authors measured a transcriptome-wide profile of ribosome association with mRNAs, including the number of ribosomes per-transcript (a total of 51,819 ribosomes on 5,700 transcripts, **S3 Table** in the original paper), their RNA copy numbers (a total of 12,534 molecules) and an estimation of the fraction of actively translated RNA copies of each type. As estimations for the total number of ribosomes (187,000 ± 56,000, of which 70–85% are engaged in active translation) and RNAs (ranging from 12,500 to 60,000) in a yeast cell vary in the literature [40,58,59], we considered 3 major scenarios: (1) A “balanced” scenario, taking the number of RNAs to be in the mid-range of the above estimates (26,000 [60]), as well as taking the number of ribosomes to be in the mid-range (187,000, of which 85% actively translating); (2) A “dense” scenario, taking the number of RNAs to be in the lower range (12,500) and the number of ribosomes to be in the upper range (243,000, of which 85% actively translating); (3) A “sparse” scenario, taking the number of RNAs to be in the upper range (60,000) and the number of ribosomes to be in the lower range (131,000, of which 70% actively translating). In the first scenario, all but 194 genes converged to feasible initiation rates (that successfully induced the number of ribosomes in steady state as reported by Arava et al., after scaling), and the median initiation rate was identical to a previous study (0.09s^-1^ [41]). In the second and third scenarios 2,341 and 4 genes failed, respectively; the median initiation rate was 0.13s^-1^ and 0.02s^-1^; and the fit of the model to experimental data was generally less accurate (**S5B Fig**). The model was tested with varying termination times ranging from 1-fold to 500-fold the typical translation time of the fastest codon, and did not find significant improvement for longer termination times and therefore set it to be as fast as the fastest codon (**S5C Fig**). Ribosome size was set to be 10 codons, based on the typical footprint length of mRPFs. Results for various choices of ribosome size appear in **S5D Fig**. It can be seen that results do not change considerably in the range of 9–15 codons, with marginally better fitting with experimental data in the range of 10–12 codons. Finally, the model was tested with different footprint spacing parameters (allowing up to 0–4 codons between ribosomes on a single footprint), and found a slight increase (but no clear maximum) in correlation with Ribo-seq double footprints (dRPFs) and single footprints (mRPFs) when allowing a higher number of free codons between ribosomes within a footprint (**S5E Fig**).

Initiation rates were also fitted to Ribo-seq and RNA-seq data using the TASEP model, for the purpose of analyzing their relation with missing mRPFs and the dRPF-to-mRPF ratio (**Fig 3B**). To this end, the number of ribosomes on each transcript was estimated using the ratio of Ribo-seq (dRPF) to RNA-seq RPKM values; the number of copies of each transcript was estimated using the RNA-seq RPKM values. Ribosome and RNA quantities were then scaled to have the same total numbers as described in the first scenario above.

### Randomization

We generated simulations for 100 genomes according to each of two models. In the first model, we permuted the rates of codons globally, i.e. by changing the translation rate of all positions of a codon in the transcriptome at once. The table with permuted codon rates was not constrained by codon synonymy, hence this model may serve as a validation for the calibration of the simulation. In the second model, we kept the original codon rates, but shuffled the order of synonymous codons across the sequence of a gene. Thus, this model controls for codon order in each transcript. In both randomizations, we selected initiation rates resulting in the same average number of ribosomes on the transcript as measured in real genes.

### Functional enrichment

We utilized annotations of GO terms (accessed 04/09/15) obtained from SGD [61] and mapped them to generic slim definitions of the biological process ontology [62]. In addition, we obtained pathways from Wiki Pathways (accessed 29 April 2016) [63].

## Supporting information

S1 Text Simulation pseudo-code. (PDF)

S1 Table Simulated traffic properties. The table reports for each gene the initiation rate, mean number of ribosomes on the transcript, translation rate, translation time, delay time, the queued fraction of ribosomes (QFR), and a measure of ramp strength based on the ratio between QFR in the first 100 codons and the middle of the gene (excluding first/last 100 codons).
(XLSX)

S2 Table Gene sets with extreme traffic conditions. Top/bottom 5% of genes according to their rank and z-scores (based on shuffling of synonymous codon order).
(XLSX)

S3 Table Datasets and mapping statistics. (XLSX)

S4 Table Codon translation times. Obtained from: Zur H, Tuller T. Tracking the evolution of mRNA translation via whole cell simulation. [57].
(XLSX)

S1 Fig Sucrose gradient fractions. **(A)** Quantification of ribosome protected footprints (RPFs) according to size. The plot shows the area under the curve (AUC) for mRPF and dRPF fractions. The ratio of dRPFs to mRPFs is reported in the caption. Peaks were detected using MATLAB’s findpeaks function, and their width was determined by the positions where the curve decreases to half the height of the peak, or where the 2^nd^ derivative turns positive. **(B)** Same for Ingolia 2009 data, profile reproduced from [23]. **(C)** Same for Guydosh 2014 data, profile reproduced from [33]. **(D)** Same for Shirokikh 2017 data, profile reproduced from [35] (Figure 5 in the original paper). See also **Fig 1.**
(EPS)

S2 Fig Fig 2 repeated for the Diament et al. dataset. (EPS)

S3 Fig Fig 2 repeated for the Guydosh et al. dataset. (EPS)

S4 Fig **(A)** Partial correlations between missing mRPFs and transcript features, as reported in **Fig 3B**, for different detection thresholds. **(B)** A histogram of simulated RPFs, *i*.*e*. the number of ribosomes in the transcriptome that are isolated or part of a pair, triplet etc. when no space is allowed between ribosomes on the same footprint. The simulated dRPF to mRPF ratio is reported. **(C)** Same, where the space of ribosomes in a footprint does not exceed 1 codons. **(D)** Same, where the space of ribosomes in a footprint does not exceed 2 codons. **(E)** Same, where the space of ribosomes in a footprint does not exceed 3 codons. **(F)** Same, where the space of ribosomes in a footprint does not exceed 4 codons.
(EPS)

S5 Fig Methods. **(A)** Distributions of mapped double ribosome protected footprints (dRPFs) in the datasets used in this study. **(B)** Calibration of simulation parameters according to 3 scenarios for the number of ribosomes and RNA molecules in a cell (details in the Materials and Methods section). Simulated mRPFs and dRPFs were sampled to have the same number of reads as in the respective Ribo-seq dataset (an aggregation of our experiment and Guydosh et al.’s). Translational efficiency (TE) was defined as the ratio between RPKM values of Ribo-seq and RNA-seq reads, and compared to simulated translation rates. Initiation rates were inferred as described in the methods and compared to data from Ciandrini et al. **(C)** Calibration of the termination rate relative to the fastest codon. **(D)** Calibration of ribosome size. **(E)** Results for the simulated RPFs for different allowed spacing within a ribosome footprint.
(EPS)
