# Supplementary material for: The extent of ribosome queuing in budding yeast
Source: PLoS Comput Biol. 2018 Jan 29;14(1):e1005951. doi: 10.1371/journal.pcbi.1005951 (PMC5805374; doi:10.1371/journal.pcbi.1005951)
Supplement: S1 Text — (PDF) [file pcbi.1005951.s001.pdf]

# The extent of ribosome queuing in budding yeast: S1 Text

## TASEP simulation

The following is a summary of the algorithm for sampling from the TASEP model (see also its description in the Materials and Methods section). In the first phase, 100 terminations are simulated in order to bring the RNA to steady state. Next, 10,000 additional terminations are simulated while monitoring relevant features, such as ribosome density.

1. Parameters:
    1.1. $\lambda$ – initiation rate.
    1.2. $\{\lambda_i\}_{i=1}^{N}$ – elongation rates of codons.
    1.3. $riboSize$ – size of ribosome in codons.
    1.4. $footSpace$ – maximal allowed codons between adjacent ribosomes within a footprint.
    1.5. $simTerms$ - number of terminations to simulate.
2. Initialize:
    2.1. $T \leftarrow 0$ (time).
    2.2. $R \leftarrow \emptyset$ (translating ribosome list).
    2.3. $term \leftarrow 0$ (termination counter).
    2.4. Monitored features:
        - $riboDensity[i] \leftarrow 0, \quad 1 \leq i \leq N$ (mean ribosome occupancy per position).
        - $footDensity[i,k] \leftarrow 0,\ 1 \leq i \leq N,\ 1 \leq k \leq N/riboSize$ (ribosome footprint density, per position and footprint size).
        - $queueDensity[i] \leftarrow 0, \quad 1 \leq i \leq N$ (mean queued ribosomes per position).
        - $transTime \leftarrow 0$ (mean translation time of the RNA).
        - $queueTime \leftarrow 0$ (mean delay time during translation).
3. Draw a time step:
    3.1. Let $\mu \leftarrow \lambda + \sum_{i=1}^{N} n_i \lambda_i$.
    3.2. Sample $dT \sim \mathrm{Exp}(\mu)$.
4. Update monitored features by integrating over $dT$:
    4.1. Increment $T$ by $dT$.
    4.2. For each ribosome $j$:
        - Increment $R[j].transTime$ by $dT$.
        - Increment $riboDensity[R[j].position]$ by $dT$.
        - If $R[j].queued$, then:
            - Increment $R[j].queueTime$ by $dT$.
            - Increment $queueDensity[R[j].position]$ by $dT$.
    4.3. For each position $i$:
        - For each footprint size $k$:
            - If position $i$ is occupied by the first ribosome in a footprint of size $k$, then increment $footDensity[i,k]$ by $dT$.
5. Sample a single event stochastically:
    5.1. Initiate with probability $\lambda/\mu$:
        - If $|R| = 0$ or $R[1].position > riboSize$, then
            - Insert a new ribosome into $R$ with the following attributes:
                - $position$: 1
                - $queued$: $FALSE$
                - $transTime$: 0
                - $queueTime$: 0
    5.2. Elongate with probability $n_i\lambda_i/\mu, \quad 1 \leq i < N$:

- Let $j$ be the index of the ribosome at site $i$.
- If $j$ is the last (3′-most) ribosome or $(R[j+1].position - R[j].position) > riboSize$, then:
  - Increment $R[j].position$.
  - $R[j].queued \leftarrow FALSE$.
- Else:
  - $R[j].queued \leftarrow TRUE$.

5.3. Terminate with probability $n_N \lambda_N / \mu$:

- $transTime \leftarrow transTime + R[end].transTime$
- $queueTime \leftarrow queueTime + R[end].queueTime$
- Remove last ribosome from $R$.
- Increment $term$.

6. If $(term < simTerms)$, then go to 3.
7. Return:

7.1. $rate \leftarrow term/T$. (mean translation rate)

7.2. $riboDensity[i] \leftarrow riboDensity[i]/T, \quad 1 \leq i \leq N$.

7.3. $footDensity[i,k] \leftarrow footDensity[i,k]/T, \quad 1 \leq i \leq N, \quad 1 \leq k \leq N/riboSize$.

7.4. $queueDensity[i] \leftarrow queueDensity[i]/T, \quad 1 \leq i \leq N$.

7.5. $transTime \leftarrow transTime/term$.

7.6. $queueTime \leftarrow queueTime/term$.
